# Supplementary material for: Waning and boosting of antibody Fc-effector functions upon SARS-CoV-2 vaccination
Source: Nat Commun. 2023 Jul 13;14:4174. doi: 10.1038/s41467-023-39189-8 (PMC10345146; doi:10.1038/s41467-023-39189-8)
Supplement: Supplementary file 1 — Supplementary Information [file 41467_2023_39189_MOESM1_ESM.pdf]

Title: Waning and boosting of antibody Fc-effector functions upon SARS-CoV-2 vaccination

Authors: Tong, X. [1, #], McNamara, R.P. [1, #], Avendaño, M.J. [2], Serrano, E.F. [2], García-Salum, T. [2,3,4], Pardo-Roa, C. [2,3], Bertera, H.L. [1], Chicz, T.M. [1], Levican, J. [2], Poblete, E. [2], Salinas, E. [2,3], Muñoz, A. [2], Riquelme, A. [3,5], Alter, G. [1, *], Medina, R.A. [2,3,4,6,*]

Affiliations:

[1] Ragon Institute of MGH, MIT, and Harvard, Cambridge, MA, 02139, USA

[2] Department of Pediatric Infectious Diseases and Immunology, School of Medicine, Pontificia Universidad Católica de Chile, Santiago 8331010, Chile

[3] Advanced Interdisciplinary Rehabilitation Register (AIRR) - COVID-19 Working Group, Faculty of Medicine, Pontificia Universidad Católica de Chile, Santiago 8331010, Chile

[4] Department of Pathology and Laboratory Medicine, School of Medicine, Emory University, Atlanta, GA 30322, USA

[5] Department of Gastroenterology, School of Medicine, Pontificia Universidad Catolica de Chile, Santiago 8331010, Chile

[6] Department of Microbiology, Icahn School of Medicine at Mount Sinai, New York, NY 10029, USA

# These authors contributed equally to this work.

* Correspondence: galter@mgh.harvard.edu and rafael.medina@emory.edu

Supplementary Information

Includes Supplementary Titles and Legends for Supplementary Figures 1 – 6.

Supplementary Information Titles and Legends

Supplementary Figure 1

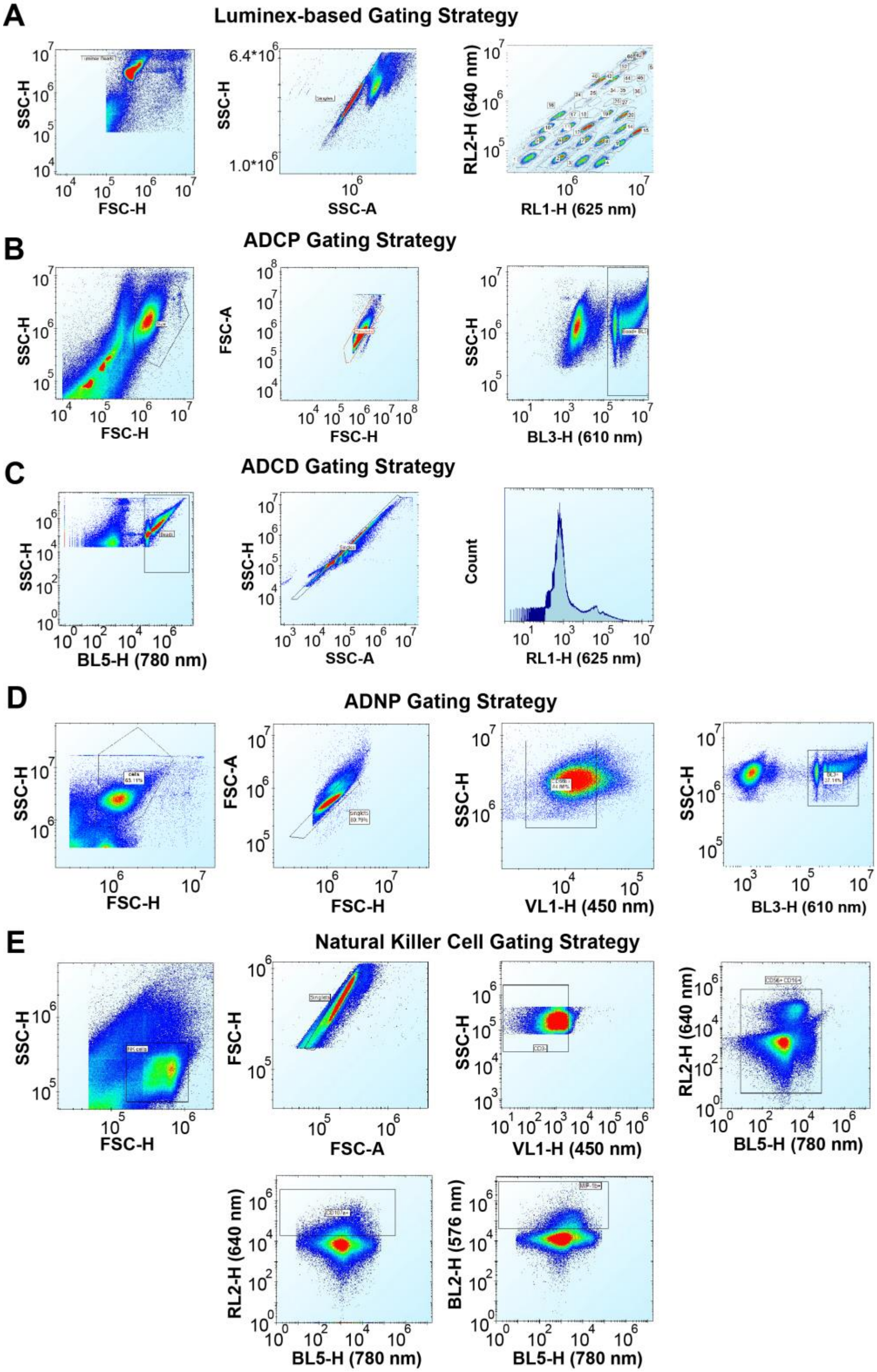

Supplementary Figure 1. Representative graphic illustration of gating strategy used to determine the FACS parameters for Systems Serology assay, **including** (A) Luminex assay, (B) Antibody Dependent Cellular Phagocytosis, (C) Antibody Dependent Complement Deposition, (D) Antibody Dependent Neutrophil Phagocytosis, (E) Natural Killer Cell Activation Assay.

Supplementary Figure 2

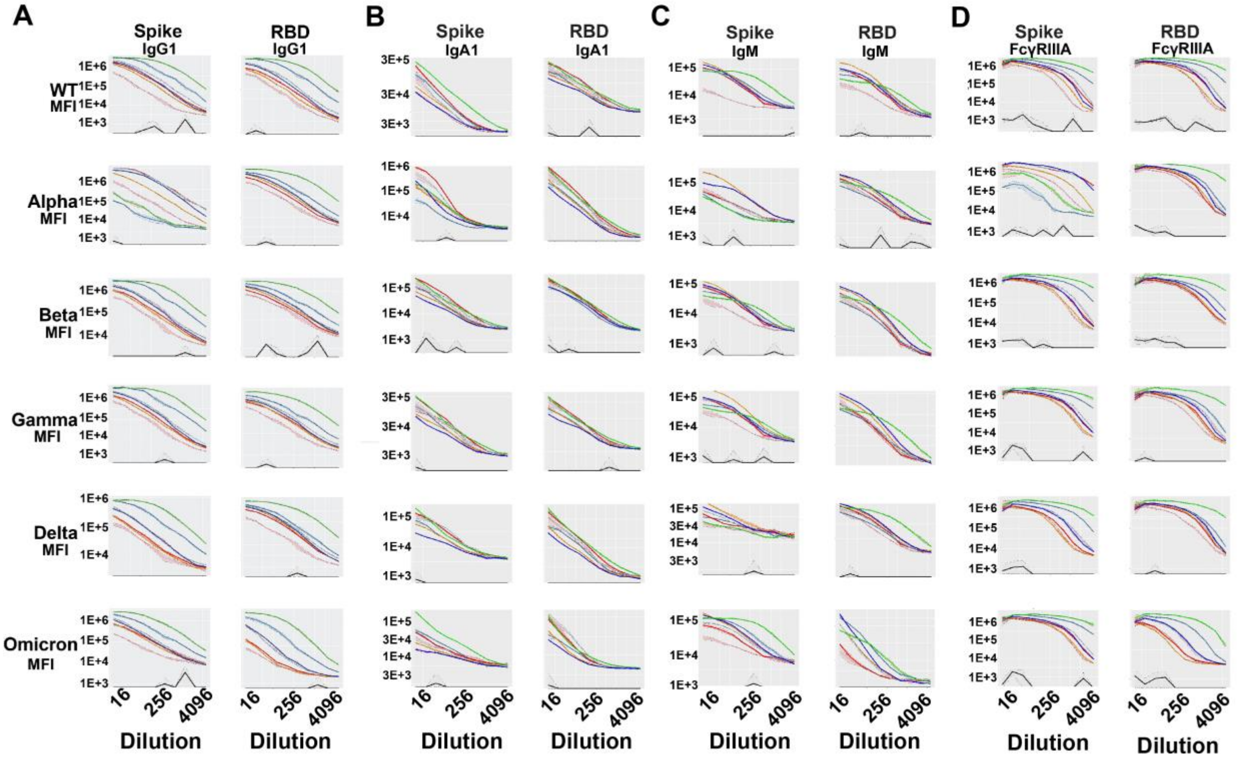

Supplementary Figure 2. Dilution curves for the antibody isotyping and receptor binding capacity. The linear ranges of the serum dilution factors were determined for (A) IgG1, (B) IgA1, (C) IgM, and (D) FcγRIIIA (IIIAV). Samples from each subgroup were randomly selected and assayed against the Wild-type, Alpha, Beta, Gamma, Delta, and Omicron variants. Each colored line represents a dilution curve for the randomly selected serum sample, whereas the black line represents the PBS control curve. All samples were assayed in technical duplicates.

Supplementary Figure 3

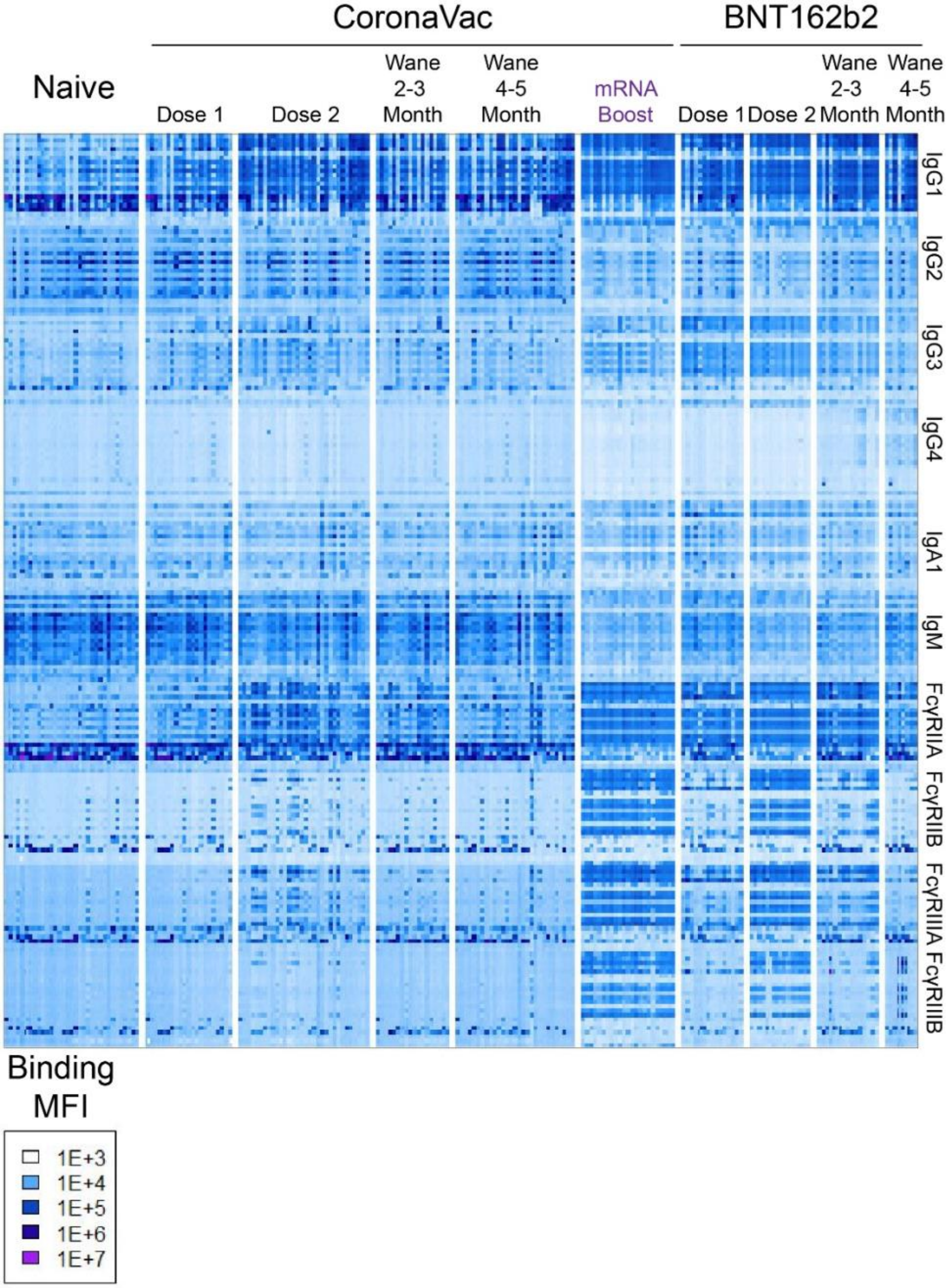

Supplementary Figure 3. Heatmap representation of binding for full-length Spike by antibody-subclasses and -isotypes and Fcγ-receptor complexes. Shown on the left are samples from the untreated naïve group (Lane 1), the CoronaVac samples from the treatments of CoronaVac doses (Lanes 2 and 3), the subsequent waning periods (Lanes 4 and 5), and the mRNA-vaccine booster (Lane 6). Shown on the right are samples under the BNT162b2 from the treatments of doses of BNT162b2 vaccines (Lanes 7 and 8) and the subsequent waning periods (Lanes 9 and 10). The scale bar is shown at the bottom of the heatmap and represents MFI over baseline values. All samples were assayed in technical duplicates.

Supplementary Figure 4

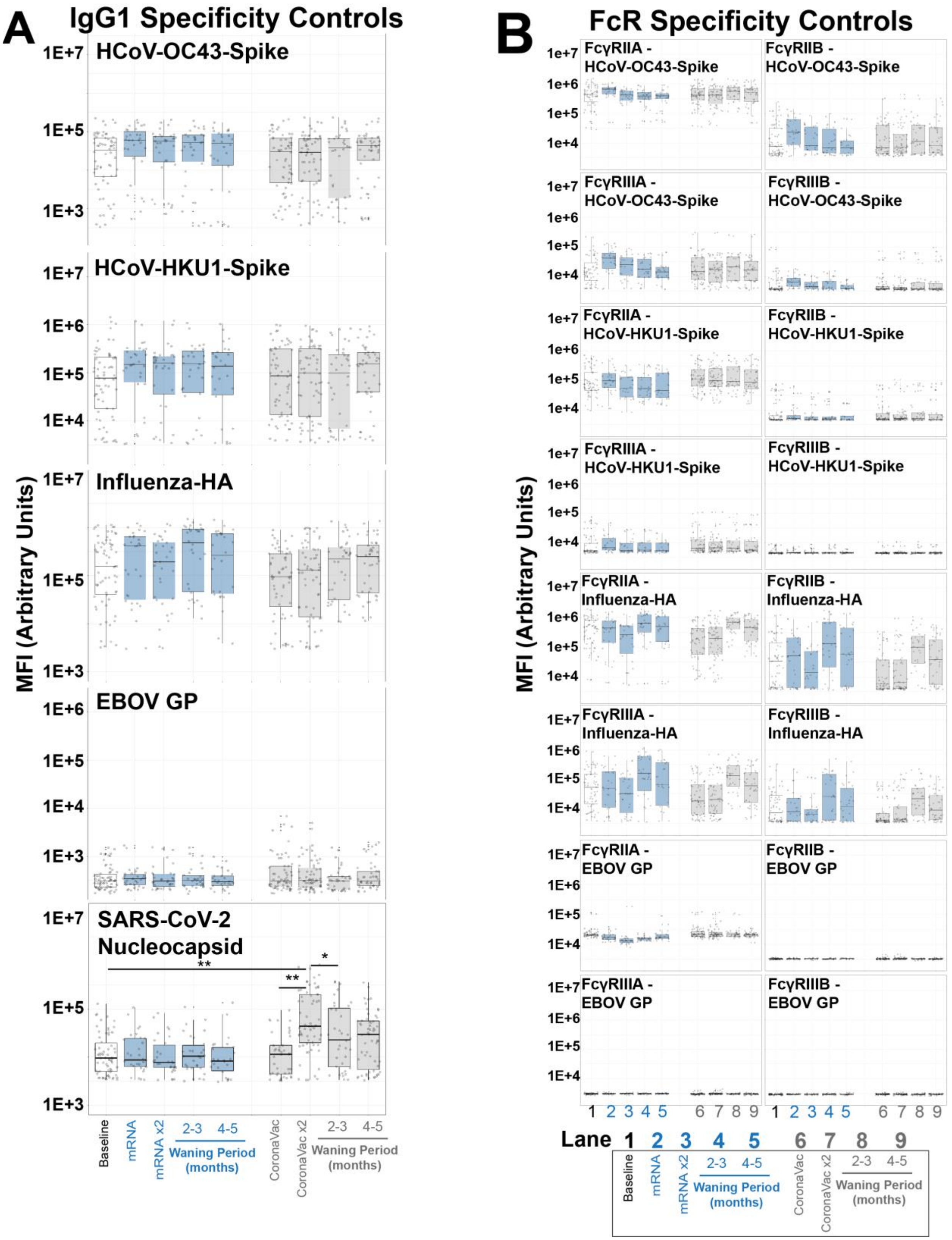

Supplementary Figure 4. COVID-19 vaccines do not have off-target humoral activation. (A) IgG1 specificity controls were quantified in the baseline (prior to immunization) (white, Lane 1), 1- and 2-dose BNT162b2 mRNA (blue, lanes 2 and 3, waning periods in lanes 4 and 5), 1- and 2-dose CoronaVac (gray, lanes 6 and 7, waning periods in lanes 8 and 9) via Luminex systems serology. Y-axis represents the mean fluorescence intensity (MFI) of a specific antigen. Shown are box and whiskers, along with individual data points, which represent the mean of individual participants from each vaccine group (BNT162b2, n = 15 and CoronaVac, n = 34). The whisker above the box plot extends from the top quartile to the highest actual value that is within the 75th percentile + 1.5 * interquartile range. The whisker below the box plot extends from the lower quartile to the lowest actual value that is within 25th percentile + 1.5 * interquartile range. Kruskal-Wallis test was used for all panels (* = $p < 0.05$ and ** = $p < 0.01$). All Kruskal-Wallis tests were two-sided, and no adjustments were made for multiple comparisons. Antigens used were against human coronavirus OC-43 (HCoV-OC43) Spike, HCoV-HKU1 Spike, Influenza HA, and Ebola virus glycoprotein (EBOV GP). The SARS-CoV-2 nucleocapsid was also included as a control. (B) Same as for A, but for FcγRIIA, FcγRIIB, FcγRIIIA, and FcγRIIIB binding for each control antigen. Lanes are shown and their corresponding products are shown at the bottom of the panel. All samples were assayed in technical duplicates.

Supplementary Figure 5

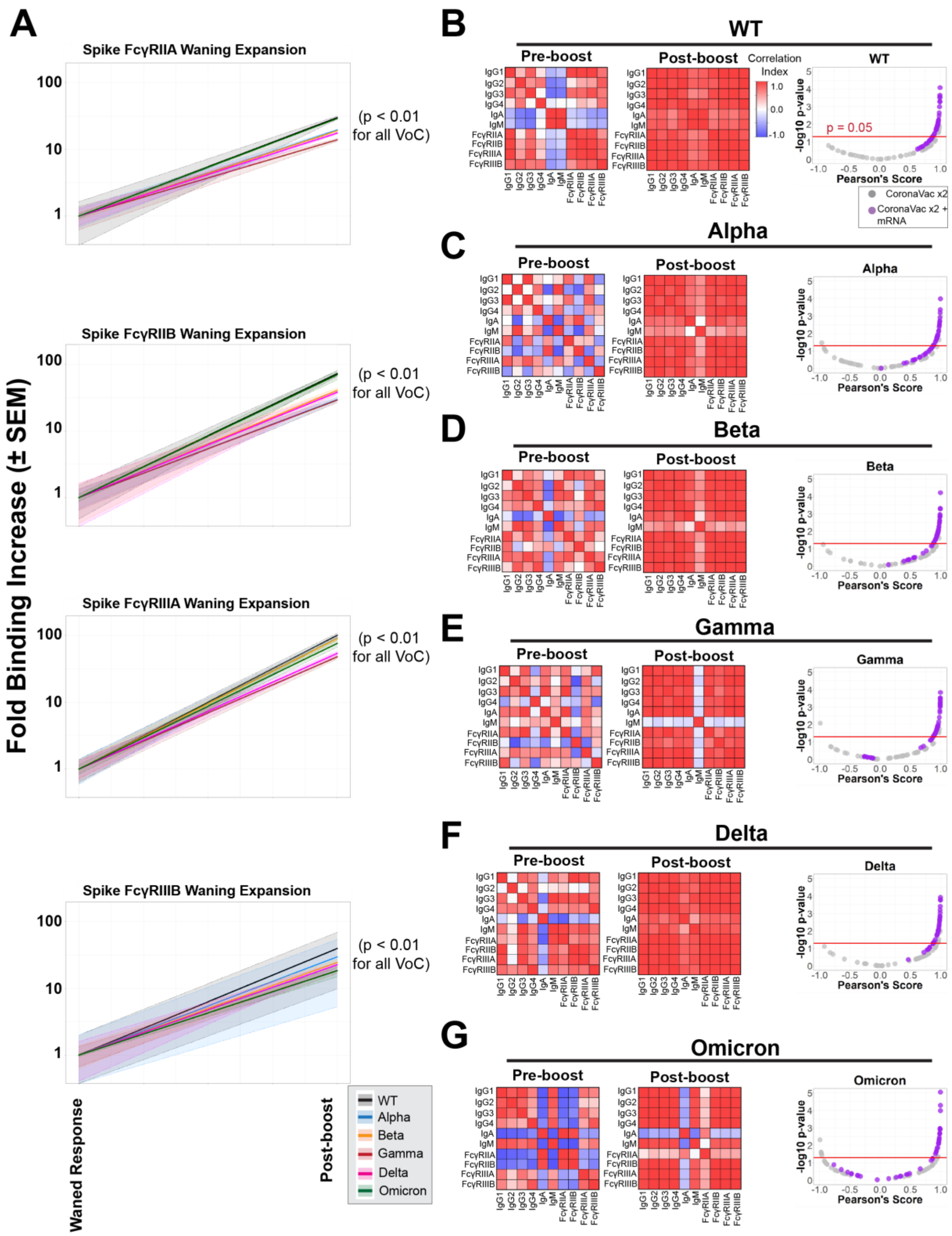

Supplementary Figure 5. mRNA-vaccine boosting significantly enhances biophysical humoral recognition against VOC Spikes. (A) Fold increases from 5-month wane windows (lane 1) to post-mRNA-vaccine boost (lane 2) for all VOC for FcRs are shown at the bottom right is the color legend for the VOC. Means were calculated using Luminex serology for each FcR for each VOC by timeframe. (B) Correlation heatmaps for WT Spike before and after mRNA-vaccine boost are shown; on the upper-right is the heatmap legend. On the right is a volcano plot of Pearson's Coefficients (x-axis) and p-values (y-axis) of pairwise antibody and Fcγ-receptor correlations of WT Spike in two-dose CoronaVac recipients (gray) and two-dose CoronaVac recipients boosted with an mRNA vaccine BNT162b2 (purple) showing a general trend towards a more tightly, and statistically significant, overall humoral response. (C – G) Same as (B), but for (C) Alpha VOC, (D) Beta VOC, (E) Gamma VOC, (F) Delta VOC, and (G) Omicron VOC. All samples were assayed in technical duplicates. All statistical tests performed were two-sided, and no adjustments were made for multiple comparisons.

Supplementary Figure 6

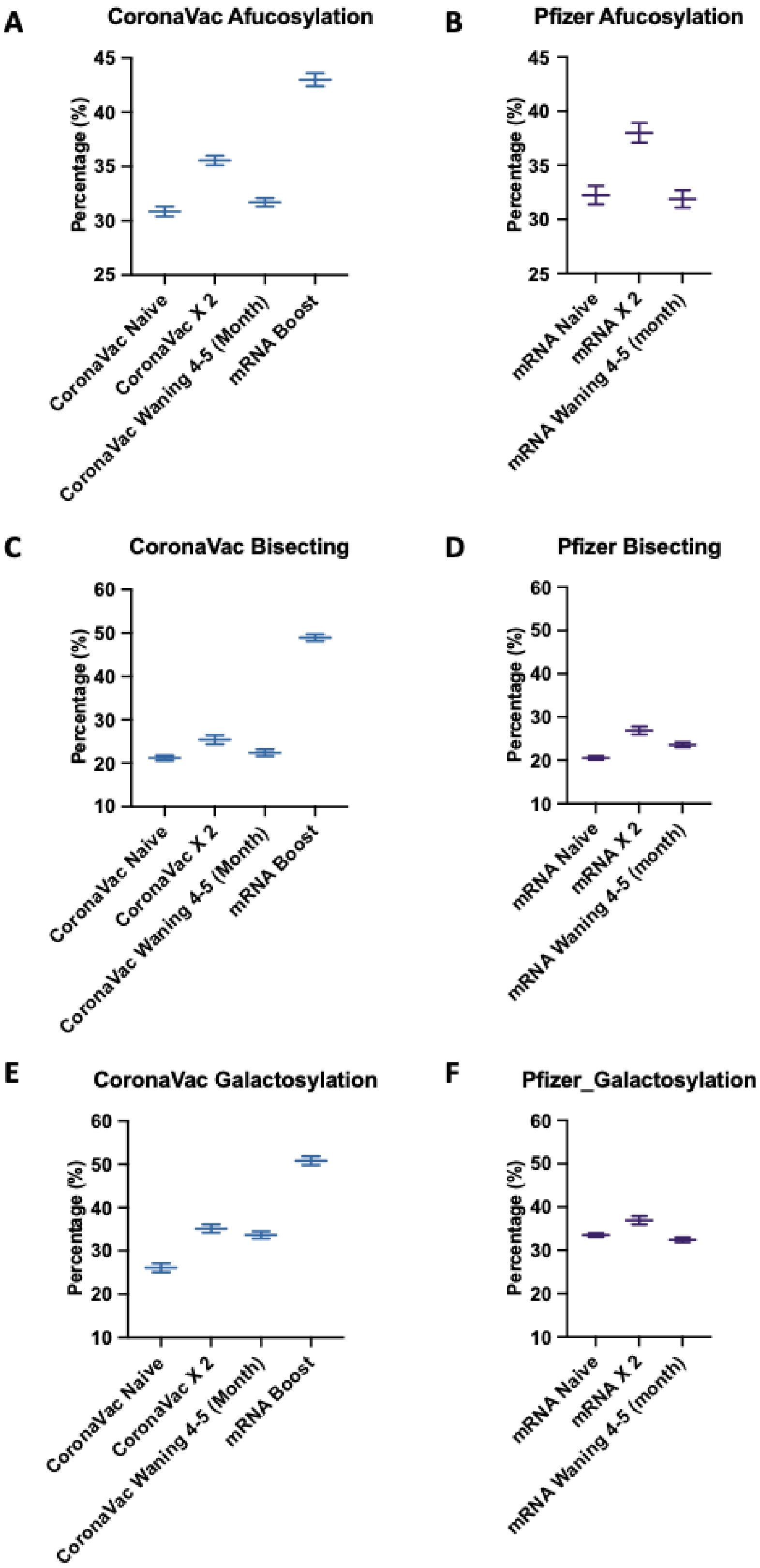

Supplementary Figure 6. Fc afucosylation levels of anti-S IgG1 are significantly reduced in vaccinated and heterologous boosted individuals. (A-B) Fc afucosylation, (C-D) bisection, and (E-F) galactosylation levels of the anti-S total IgG1 from individuals receiving the first regimen of two doses of CoronaVac (A, C, E) or BNT162b2 (B, D, F) and subsequently the heterologous boosting was assayed. Samples from pre-vaccination (naïve), peak immunity (CoronaVac or mRNA X2), the lowest level during the waning period (CoronaVac or mRNA Waning 4-5 Month), and after heterologous boosting (mRNA Boost) were combined and analyzed through electrophoresis. All samples were assayed in technical duplicates. The central line represents the median value of individual samples pooled from each vaccine group (BNT162b2, n = 10 and CoronaVac, n = 10), where the whiskers extend down to the smallest value and up to the largest value for each box.
