# Supplementary material for: Waning and boosting of antibody Fc-effector functions upon SARS-CoV-2 vaccination
Source: Nat Commun. 2023 Jul 13;14:4174. doi: 10.1038/s41467-023-39189-8 (PMC10345146; doi:10.1038/s41467-023-39189-8)
Supplement: Supplementary file 3 — Reporting Summary [file 41467_2023_39189_MOESM3_ESM.pdf]

nature portfolio

Corresponding author(s): Galit Alter, Rafael Medina

Last updated by author(s): May 15, 2023

Reporting Summary

Nature Portfolio wishes to improve the reproducibility of the work that we publish. This form provides structure for consistency and transparency in reporting. For further information on Nature Portfolio policies, see our Editorial Policies and the Editorial Policy Checklist.

Statistics

For all statistical analyses, confirm that the following items are present in the figure legend, table legend, main text, or Methods section.

n/a	Confirmed	
☐	☒	The exact sample size (*n*) for each experimental group/condition, given as a discrete number and unit of measurement
☐	☒	A statement on whether measurements were taken from distinct samples or whether the same sample was measured repeatedly
☐	☒	The statistical test(s) used AND whether they are one- or two-sided<br>*Only common tests should be described solely by name; describe more complex techniques in the Methods section.*
☐	☒	A description of all covariates tested
☐	☒	A description of any assumptions or corrections, such as tests of normality and adjustment for multiple comparisons
☐	☒	A full description of the statistical parameters including central tendency (e.g. means) or other basic estimates (e.g. regression coefficient) AND variation (e.g. standard deviation) or associated estimates of uncertainty (e.g. confidence intervals)
☐	☒	For null hypothesis testing, the test statistic (e.g. *F*, *t*, *r*) with confidence intervals, effect sizes, degrees of freedom and *P* value noted<br>*Give P values as exact values whenever suitable.*
☒	☐	For Bayesian analysis, information on the choice of priors and Markov chain Monte Carlo settings
☒	☐	For hierarchical and complex designs, identification of the appropriate level for tests and full reporting of outcomes
☐	☒	Estimates of effect sizes (e.g. Cohen's *d*, Pearson's *r*), indicating how they were calculated

Our web collection on statistics for biologists contains articles on many of the points above.

Software and code

Policy information about availability of computer code

Data collection	Data was collected using the IQue Screener Plus using the iQue Forecyt 9.1 visualization and analysis package. All flow cytometry data was exported into individual .csv files.
Data analysis	Data was analyzed and plotted using R Studio v 1.4.1103, GraphPad Prism v 9.3.1. No new code was generated in this manuscript.

For manuscripts utilizing custom algorithms or software that are central to the research but not yet described in published literature, software must be made available to editors and reviewers. We strongly encourage code deposition in a community repository (e.g. GitHub). See the Nature Portfolio guidelines for submitting code & software for further information.

Data

Policy information about availability of data

All manuscripts must include a data availability statement. This statement should provide the following information, where applicable:

- Accession codes, unique identifiers, or web links for publicly available datasets
- A description of any restrictions on data availability
- For clinical datasets or third party data, please ensure that the statement adheres to our policy

The Systems Serology data generated in this study have been deposited in the ImmPort database under accesion code SDY2216. Further information and requests for primary datasets, resources, and/or reagents should be directed to and will be fulfilled by the lead data point of contact, Ryan P. McNamara (rpmcnamara@mgh.harvard.edu), or the corresponding authors, Galit Alter (galter@mgh.harvard.edu), or Rafael A. Medina (rafael.medina@emory.edu). Any

additional information required to reanalyze the data reported in this paper is available from the lead contact upon request within a reasonable time frame. All codes and scripts are available upon request to the lead data point of contact. We request that downstream data reuse reference the manuscript and acknowledge the corresponding authors.

Human research participants

Policy information about studies involving human research participants and Sex and Gender in Research.

Reporting on sex and gender	Findings in the manuscript apply to both sexes as both male and female participants were analyzed. A brief description of the sexes and age demographics of the individuals in this study is included in the manuscript and in the section below. All data analysis was blinded in regards to sex.
Population characteristics	The cohort contains samples from individuals who received either BNT162b2 (n = 15) or CoronaVac vaccines (n = 34). The BNT162b2 mRNA vaccine group was given 30 µg BNT162b2 (23 – 53 years old, median: 36 years, 81% female) on days 0 and 21, and serum samples were taken up to 209 days after the second dose. The CoronaVac group (23 – 46 years old, median: 31 years old, 61% female) received two doses of 600 U CoronaVac four weeks apart and individuals were sampled 1-209 days after the second dose.
Recruitment	Healthy individuals that received their COVID-19 vaccine and booster immunizations at the UC-Christus Health network in Santiago, Chile, were invited to participate in the study. There was no specific selection of participants and they represent the overall population vaccinated in the UC-Christus Health Network between January and September 2023. An informed written consent form was obtained under protocol 200829003, which was reviewed and approved by the Health Sciences Scientific Ethics Committee at Pontificia Universidad Católica de Chile (PUC).
Ethics oversight	The Health Sciences Scientific Ethics Committee at Pontificia Universidad Católica de Chile (PUC) reviewed and approved this study (protocol 200829003). This work was supervised and approved by the Mass General Institutional Review Board (IRB #2020P00955 and #2021P002628).

Note that full information on the approval of the study protocol must also be provided in the manuscript.

Field-specific reporting

Please select the one below that is the best fit for your research. If you are not sure, read the appropriate sections before making your selection.

☒ Life sciences ☐ Behavioural & social sciences ☐ Ecological, evolutionary & environmental sciences

For a reference copy of the document with all sections, see nature.com/documents/nr-reporting-summary-flat.pdf

Life sciences study design

All studies must disclose on these points even when the disclosure is negative.

Sample size	The cohort analyzed represent individuals that received the primary two-dose CoronaVac vaccine series (n = 34) or the two-dose mRNA BNT162b2 vaccine series (n = 15). A subset of CoronaVac recipients also received an mRNA BNT162b2 vaccine booster (n = 20). For all analysis, technical and biological replicates were performed for each sample. This was an opportunistic sampling of individuals receiving their COVID-19 vaccine and booster immunizations at the UC-Christus Health network in Santiago, Chile. This is an observational study and no sample size was predetermined. Nonetheless, from the COVID-19 literature and from studies previously conducted by our group with this same cohort to look at neutralizing antibodies (EBioMedicine. 78:103972. DOI: 10.1016/j.ebiom.2022.103972), we determined that statistically significant data can be obtained for serological data analyses of this study participants.
Data exclusions	No data was excluded from this manuscript.
Replication	Independent, biological replicates for each assay were performed to ensure reproducibility yielding similar results.
Randomization	Individuals were assigned to groups based on the vaccine series, CoronaVac or BNT162b2. The boosted group inhabited their own grouping,
Blinding	The investigators who performed the experiments were blinded before and while conducting the reported experiments. The samples were randomized for all experimental procedures and the investigators were unblinded only after the data were collected.

Reporting for specific materials, systems and methods

We require information from authors about some types of materials, experimental systems and methods used in many studies. Here, indicate whether each material, system or method listed is relevant to your study. If you are not sure if a list item applies to your research, read the appropriate section before selecting a response.

Materials & experimental systems

n/a	Involved in the study	
☐	☒	Antibodies
☐	☒	Eukaryotic cell lines
☒	☐	Palaeontology and archaeology
☒	☐	Animals and other organisms
☐	☒	Clinical data
☒	☐	Dual use research of concern

Methods

n/a	Involved in the study	
☒	☐	ChIP-seq
☐	☒	Flow cytometry
☒	☐	MRI-based neuroimaging

Antibodies

Antibodies used

Anti-human IgG1-PE (Southern Biotech HP6001), IgG2-PE Z(31-7-4), IgG3-PE (HP6050), IgG4-PE (HP6025), IgM-PE (SA-DA4), and IgA1 (HP6025) were all obtained from Southern Biotech. These antibodies were used for systems serology biophysical charaterizations and quantitations. AntiCD66b Pac Blu (BioLegend 305112), CD107a (BD Biosciences, 555802), CD3 (BD Biosciences, 558117), CD16 (BD Biosciences, 557758), CD56 (BD Biosciences, 557747), IFN-gamma (BD Biosciences, 340449), CCL4 (BD Biosciences, 550078), C3b (MP Biomed 855385), Anti-human IgG1-PE (Southern Biotech, 9054-09), IgG2-PE (Southern Biotech, 9040-09), IgG3-PE (Southern Biotech, 9210-09), IgG4-PE (Southern Biotech, 9190-09), IgM-PE (Southern Biotech, 9022-09), and IgA1-PE (Southern Biotech, 9130-09) were used for immunological functional asssays and gating of specific cell types.

Validation

All antibodies used have been previously validated in the following manuscripts previously published by our group: PMID 33589825, 34652962, 33589636, 35325158, 35881018, 35430229, 35090580, and 35289637. All of these manuscripts have been peer-reviewed.

Eukaryotic cell lines

Policy information about cell lines and Sex and Gender in Research

Cell line source(s)

THP-1 monocyte cell was obtained from the ATCC (TIB-202).

Authentication

Authentication was provided by the manufacturer (e.g morphology)

Mycoplasma contamination

Cell line was validated to be mycoplasma free.

Commonly misidentified lines
(See ICLAC register)

No commonly misidentified cell lines were used in the study

Clinical data

Policy information about clinical studies

All manuscripts should comply with the ICMJE guidelines for publication of clinical research and a completed CONSORT checklist must be included with all submissions.

Clinical trial registration

This study was not a clinical trial.

Study protocol

Health Sciences Scientific Ethics Committee at Pontificia Universidad Católica de Chile 200829003. The work was supervised and approved by the MGH IRB protocols 2020P00955 and 2021P002628.

Data collection

Patients were recruited and enrolled in the time period of 01/2021 - 09/2021.

Outcomes

Clinical outcomes were not reported in this manuscript.

Flow Cytometry

Plots

Confirm that:

☒ The axis labels state the marker and fluorochrome used (e.g. CD4-FITC).

☒ The axis scales are clearly visible. Include numbers along axes only for bottom left plot of group (a 'group' is an analysis of identical markers).

☒ All plots are contour plots with outliers or pseudocolor plots.

☒ A numerical value for number of cells or percentage (with statistics) is provided.

Methodology

Sample preparation

Sera was obtained and stored at -80C until use. Sera was diluted in 1X PBS at 1:100 (for IgG2, IgG3, IgG4, IgM, and IgA1),

Sample preparation	1:500 for IgG1, and 1:750 for all Fc-gamma receptor binding.
Instrument	For flow cytometry, all samples were run on the iQue Screener Plus (Intellicyt/Sartorius 11811). 384 plates were washed using the 384-well HydroSpeed Plate Washer (Tecan 30190112).
Software	Flow cytometry plots were analyzed using the iQue Forcyt (Sartorius 60028) and FlowJo V 10.8. Subsequent analysis was done using R Studio V 1.4.1103 and GraphPad Prism V 9.3.1.
Cell population abundance	No sorting was done in this manuscript.
Gating strategy	The gating strategy used in this manuscript is the same as previously validated and peer-reviewed articles: PMID 33589825, 34652962, 33589636, 35325158, 35881018, 35430229, 35090580, and 35289637.

☒ Tick this box to confirm that a figure exemplifying the gating strategy is provided in the Supplementary Information.
